# Fine Mapping of a QTL for Fertility on BTA7 and Its Association With a CNV in the Israeli Holsteins

**DOI:** 10.1534/g3.111.000299

**Published:** 2011-06-01

**Authors:** Giora Glick, Andrey Shirak, Eyal Seroussi, Yoel Zeron, Efraim Ezra, Joel I. Weller, Micha Ron

**Affiliations:** * The Hebrew University of Jerusalem, The Robert H. Smith Faculty of Agriculture, Rehovot, 76100, Israel; † Institute of Animal Sciences, ARO, The Volcani Center, Bet Dagan 50250, Israel; ‡ Sion, AI Institute, Shikmim 79800, Israel; § Israel Cattle Breeders Association, Caesaria Industrial Park, Caesaria 38900, Israel

**Keywords:** quantitative trait locus (QTL), copy number variation (CNV), *KIAA1683*, gene-variants, female-fertility

## Abstract

A quantitative trait locus (QTL) affecting female fertility, scored as the inverse of the number of inseminations to conception, on *Bos taurus* chromosome 7 was detected by a daughter design analysis of the Israeli Holstein population (*P* < 0.0003). Sires of five of the 10 families analyzed were heterozygous for the QTL. The 95% confidence interval of the QTL spans 27 cM from the centromere. Seven hundred and four SNP markers on the Illumina BovineSNP50 BeadChip within the QTL confidence interval were tested for concordance. A single SNP, NGS-58779, was heterozygous for all the five QTL heterozygous patriarchs, and homozygous for the remaining five QTL homozygous sires. A significant effect on fertility was associated with this marker in the sample of 900 sires genotyped (*P* < 10^−6^). Haplotype phase was the same for four of the five segregating sires. Thus concordance was obtained in nine of the ten families. We identified a common haplotype region associated with the rare and economically favorable allele of the SNP, spanning 270 kbp on BTA7 upstream to 4.72 Mbp. Eleven genes found in the common haplotype region should be considered as positional candidates for the identification of the causative quantitative trait nucleotide. Copy number variation was found in one of these genes, *KIAA1683*. Four gene variants were identified, but only the number of copies of a specific variant (V_1_) was significantly associated with breeding values of sires for fertility.

Poor reproductive performance is one of the most common reasons for premature culling. Low female fertility causes economic losses by increasing the need for additional inseminations, higher veterinary costs, longer than optimal lactations, less calves, and higher replacement costs. Therefore, improving female fertility is important for dairy cattle breeders. Various statistics are used to measure fertility of dairy cattle in different countries. In Israel, fertility is scored as the inversed number of inseminations to conception. Heritability for nearly all measures of fertility is only 1–5% (Gonzalez-Recio *et al.* 2006; Kuhn *et al.* 2006; Weller and Ezra 1997), and has negative genetic correlations with milk production traits (Gonzalez-Recio *et al.* 2006; Veerkamp *et al.* 2001; Weller and Ezra 1997).

Weller *et al.* (2008) conducted a daughter design QTL scan for economic traits on BTA7 in Israeli Holstein population. The scan included genotyping 29 microsatellites spanning the chromosome in 11 sires families. Two significant effects for female fertility QTL were identified; near the centromere with five segregating sire families, and near the end of the chromosome with two segregating sire families. The 95% confidence intervals (CI) of the QTL near the centromere spans 27 cM, which includes hundreds of genes.

The most convincing methodology for identifying the causative polymorphism for segregating QTL, the quantitative trait nucleotide (QTN), is concordance (Ron and Weller 2007). Complete concordance is obtained only if:

1. All individuals known to be homozygous for the QTL are also homozygous for the polymorphism.
2. All individuals heterozygous for the QTL are also heterozygous for the polymorphism.
3. The same QTL allele is associated with the same allele of the putative QTN for all the heterozygous animals.

Testing for concordance requires determination of the QTL genotype of several individuals, which can be determined for family patriarchs by either a daughter or granddaughter design (Ron and Weller 2007). The first two requirements for concordance can be analyzed directly from daughter or granddaughter designs results. The third requirement for concordance requires the identification of the QTL phase of the segregating sires. Concordance can be considered as a proof for QTN detection if the probability of obtaining concordance by chance is sufficiently low for rejection of this hypothesis (Ron and Weller 2007). For a given number of patriarchs with known QTL genotype, probability of obtaining concordance by chance decreases as the fraction of heterozygous sires increases (Weller *et al.* 2008). Therefore, we focused the analysis on the QTL near the centromeric region of BTA7, because five sires were heterozygous for this QTL.

Recently, 900 Israeli Holstein sires with genetic evaluations for milk production traits and female fertility, including the 10 patriarchs of the daughter design, were genotyped for the Illumina BovineSNP50 BeadChip, which includes 54,001 SNPs. The aim of this study was to identify SNPs with significant concordance within the CI of the female fertility QTL in the centromeric region of BTA7, and to use this data to identify likely candidate genes for the QTL.

## Materials and Methods

### Illumina BovineSNP50 BeadChip genotyping

DNA samples of 900 Israeli Holstein bulls were genotyped for 54,001 SNPs using the Illumina BovineSNP50 BeadChip. Quality control and genotyping procedure are as shown by Weller *et al.* (2010).

### Concordance testing

A total of 704 SNP markers included on the Illumina BovineSNP50 BeadChip are positioned within the first 30 Mbp of BTA7, which cover the CI of the QTL for female fertility. The genotypes of the 704 SNPs for the 10 sires with inferred genotypes for the QTL from the daughter design were tested for concordance, of which five were heterozygous and five were homozygous for the QTL.

The SNP marker that showed the highest fit to the concordance model is NGS-58779. All five sires, homozygous for the QTL, were homozygous for the marker and all five patriarchs, heterozygous for the QTL were heterozygous for the marker. Nevertheless, the haplotype phase was concordant for only four of the five segregating sires. Thus concordance was obtained in 9 out of the 10 families. The probability to obtain a genetic marker with similar concordance by chance was computed assuming that the QTL allele frequency is 0.5, as half of the families segregated for the QTL. Assuming independence among the sire genotypes, the probability by chance that the five sires homozygous for the QTL will also be homozygous for the marker is 0.5^5^, the probability that four sires will be heterozygous for the marker and the QTL with correct allele phase between the QTL and the marker is: 0.25^4^. The probability that one out of five heterozygous sires for the QTL will be heterozygous for the marker with incorrect allele phase between the QTL and the marker is: 0.25*5. Thus, the probability to obtain this level of concordance by chance for a single SNP is: 0.5^5^* 0.25^4^* 0.25*5 = 0.000152. As 704 SNPs were analyzed 0.1 SNP is expected to show this level of concordance by chance.

### Haplotype phase analysis

Genotypes of 20 SNP markers from the Illumina BovineSNP50 BeadChip, positioned within the flanking 0.5 Mbp upstream and downstream to NGS-58779, were analyzed for 900 Israeli Holstein sires. Haplotype phase analysis was performed using the Plink software with individuals treated as unrelated (Purcell *et al.* 2007). The resulting haplotypes of the 10 tested sires by the daughter design were verified by LSPH software that is based on known genetic relationships between individuals (Baruch *et al.* 2006).

### Comparative mapping

Comparative mapping between the bovine common haplotype region and the human orthologous region was conducted using Ensembl biomart (http://www.ensembl.org/biomart/martview) and NCBI map-viewer (http://www.ncbi.nlm.nih.gov/projects/mapview/).

### Positional cloning

A partial screen of the exons of six of the 11 genes within the identified common haplotype region (*KIAA1683*, *PDE4C*, *CIST1*, *JUND*, *LSM4*, and *GDF15*) was performed in order to identify concordant polymorphism for the QTL. Primers were designed by the Primer3plus software (http://www.bioinformatics.nl/cgi-bin/primer3plus/primer3plus.cgi) in accordance with the exon-intron borders obtained from the reference sequence of the NCBI Entrez Gene database (http://www.ncbi.nlm.nih.gov/gene/). DNA of sires was extracted from semen samples as described previously (Ma *et al.* 1996). The bovine *KIAA1683* genes (*LOC618787* and *LOC788637*) were sequenced simultaneously, due to the very high similarity in their predicted exonic sequences. The primers that were used for the positional cloning are presented in Table 1.

**Table 1:** PCR and sequencing primers

Gene	Forward Primer	Reverse Primer	Product Size
*KIAA1683*			
Exon 1	GTACCTGCCAAGTGGAGGAG	TGACGCCTGTAGCTGTGAAC	283
Exon 2.1	TTCACAGCTACAGGCGTCAC	CCTTGTTGGCTCTGGAAGTT	782
Exon 2.2	ACCCAATGAAGACCAAGCAA	GGCCTTGGTTATTGTGACAG	509
Exon 3	GCCTCGATGATCAAGTCTCC	AGATGTCCGTCCTCGGATGT	700
Exon 4	GAGGACGGACATCTTGAAGG	CACACAGGGAGGCGAGTT	794
Intron 4	GGTCCGTGATCAGTGGTG	ACTGCTGCTCATGGGATCTA	811
Exon 5-6.1	CCCTGACTCCAGCTGACA	CCGCCTTGGTCTGATGAT	795
Exon 6.2	CACCCCATCATCAGACCAA	CCCACCAGTAAGCCAAGGT	232
SP_1_	(T)_26_GTAAAACGACGGCCAGTAACTGCTCCTCCCCTGACa		531
SP_2_	CAGGAAACAGCTATGACAACTGCTCCTCCCCTGACg		505
*JUND*			
Exon 1.1	CCGGGCCGAGGCTATAAG	CTCTGCCTTAATGCGCTCTT	997
Exon 1.2	GTCTACGCGAACCTGAGCA	CCAACGTTTGTTCCCGAGTA	911
Exon 1.3	CTCAGACTCGACAAGCTGGA	CCCTGCAGGAGAAGAAGGAG	577
*LSM4*			
Exon 1	ATTGGTTAGCCTTCCCACGA	ATGACACACTCGCTCTGCAT	236
Exon 2	ATATGCCAGCCCTGCCTAGA	AGCTGCTGCTTCGTGGAC	250
*GDF15*			
Exon 1	CGGACAAAGTCCAGGGAATA	TGGGGTCTCCGAAATTTTAC	385
Exon 2	AGCTTTCTTTCCTCTTTTCCA	CGTCCATGCCATAAATGAAG	700
*CIST1*			
Exon 1	AACATGCGCCCCTCACAT	GCCGGATCTTACCTGTTCTT	405
Exon 2	GGGTTGCATATCCTTGTTCC	CCCCAATGTCCCAATACTCA	664
Exon 3-4	GGCCCTGGTGTGTCTGTC	GCCTCAGAGGAATGATAGGG	554
NGS-58779	TGAGCTGAGACTGGAAAGAGG	AATCCACACCCAGAAGCAAC	231
M-13	GTAAAACGACGGCCAGT	CAGGAAACAGCTATGAC	

PCR fragments were amplified using Super-Therm Taq DNA polymerase (JMR Holding, London) according to the instructions of the manufacturer and the following conditions: 30 cycles for 30 s at 92°C, 40 s at 63°C, and 1 min at 72°C, using a DNA engine thermocycler (MJ Research Inc., Waltham, MA). PCR products were separated on agarose gels, excised from the gel, purified with DNA Montage Gel Extraction Kit (Millipore, Bedford, MA). Sequencing was preformed using the BigDye Terminator v3.1 Cycle Sequencing Kit using the 3130 Genetic Analyzer capillary electrophoresis instrument (Applied Biosystems). Sequences were assembled and compared using the GAP4 program (Staden *et al.* 1999).

### Isolation of *KIAA1683* variants

Specific primers (SP) were designed to PCR amplify separately each of the A/G alleles located in *KIAA1683* at position 4856941 /4896528 bp on BTA7, which was heterozygous in all of the sires (Table 1). An “A” to “T” mismatch in the fifth nucleotide from the 5′ end of the SP primers, indicated by lower case letter, was introduced to increase the specificity of the PCR amplification. Standard M-13 phage forward or reverse primer sequences were added to the 3′ core primer sequence to serve as a template for sequencing primers and to decrease primers dimer formation. An additional 26 repeats of T tail was attached to the 3′ end of the SP_1_ primer to allow detection of fragment size difference from the SP_2_ PCR product on a 2% agarose gel. The *KIAA1683*_exon3_R primer was used as a reverse primer (Table 1). PCR products were amplified by high-fidelity BIO-X-ACT Long DNA polymerase (Bioline, London) according to the instructions of the manufacturer and the following conditions: 30 cycles for 30 s at 92°C, 40 s at 64°C, and 1 min at 72°C, using a DNA engine thermocycler (MJ Research Inc., Waltham, MA). SP_1_ and SP_2_ PCR products were sequenced using the M-13_F and R primers, respectively.

The sequences of the isolated variants of the *KIAA1683* gene were Blasted against the bovine WGS traces and the HTGS sequence databases (NCBI) for sequence validation, and to identify alternative variants of the gene which were not found among the 10 sires.

### Real-Time qPCR

Determination of the relative copy number of the total *KIAA1683* gene (including all gene variants) was conducted using quantitative real-time PCR (qPCR) analysis. A total of 326 Israeli Holstein bulls with known breeding values for fertility including the 10 sires that were tested by the daughter design were analyzed. Primers were designed on a region of the exon 3 which was common and monomorphic to all of the identified gene variants. Relative copy numbers of V_1_, V_2_, and V_3_ were determined using variant specific selective primers. V_1_ selective primer was designed to amplify the “T” allele of the 4857096/4896683 polymorphism, which is unique for V_1_. V_2_ selective primer was designed to amplify the unique “A” allele of the 4857153/4896740 polymorphism, and the V_3_ selective primer was designed to amplify the unique “T” allele of the 4857126/4896713 polymorphism. M-13 phage forward standard primers sequences were attached to the 5′ of the core primers to decrease primers dimer formation. The same reverse primer was used for both V_1_ and V_2_ selective primers. qPCR primers are shown in Table 2. V_4_ does not possess a unique polymorphism which can be used for selective qPCR.

**Table 2:** qPCR primers

Primer ID	Sequence	Product Size (bp)
*RPP30*_F	TGCTTCCATTGTTTCCTGATGA	96
*RPP30*_R	TGGGACCAGGTTCCATGATC	
Total-*KIAA*1683_F	CCACCATCCTCAAAACCCTGT	101
Total-*KIAA1683*_R	GGATGAGGTGTCGGAAATTCC	
V_1__F	GTAAAACGACGGCCAGTTTTCCGACACCTCATCCGACAT	214/155
V_2__F	GTAAAACGACGGCCAGTGAGACAGGCGACCTGGGA	
V_1_/V_2__R	GGAAACAGCTATGACATTTTCTCGCCTTCCAAAGG	
V_3__F	GTAAAACGACGGCCAGTAAATGCTTTCCCCGGTCA	121
V_3__R	CAGGAAACAGCTATGACCGTGGTTTCCGTTTTCCCTT	

Gene copy number was normalized to an autosomal reference gene, Ribonuclease P protein subunit p30 (*RPP30*, GeneID:615098, on BTA26. A fragment of 95 bp at position 12893277), *RPP30*, was used as a reference gene in human CNV studies (Wang *et al.* 2010). No CNV was reported for this gene region in previous studies of CNV in cattle (Bae *et al.* 2010; Fadista *et al.* 2010; Liu *et al.* 2010; Seroussi *et al.* 2010). The qPCR analysis was performed in duplicates using the Absolute Blue SYBER Green ROX mix kit (Thermo Fisher scientific, UK) according to the instructions of the manufacturer in a 17-µl reaction volume, which included 2 µl of DNA (30 ng/µl), 1 µl of each primer (10 pmol/µl), 7.5 µl of ultra pure water, and 7.5 µl of Absolute Blue SYBER Green ROX Mix. The qPCR reaction was performed in the following conditions: 15 min at 95°C for enzyme activation followed by 40 cycles of 15 s at 95°C, 1 min at 60°C, using an ABI Prism 7000 sequence detection system. Amplification was followed by a dissociation curve analysis to confirm the presence of a single product and the absence of primer dimer. The threshold cycle number (C_T_) for each tested probe was used to quantify its relative abundance. The qbasePLUS software (Biogazelle, Ghent, Belgium) was used for the calculation of the relative quantities using *RPP30* for normalization (D'haene *et al.* 2010). The qPCR primers that were designed to selectively amplify V_3_ failed, thus V_3_ was excluded from the analysis.

Correlations between the C_T_s of the two duplicates were 0.8, 0.8, 0.7, and 0.9 for V_1_, V_2_, total *KIAA1683*, and *RPP30*, respectively. The standard curves of all qPCR probes were linear in all the tested DNA dilutions with R^2^ <0.98 and with slopes of −3.36, −3.30, −2.99, and −3.11, which indicate efficiency of 101, 100, 93, and 92% for V_1_, V_2_, total *KIAA1683*, and *RPP30*, respectively. The average C_T_ of the lowest detectable DNA concentration point (LOD) in the standard curves were 30.7, 30.6, 26.8, and 25.7, while the average C_Ts_ of the nontemplate controls (NTC) were 37.1, 35.1, 32.8, and 31.9 for V_1_, V_2_, total *KIAA1683*, and *RPP30*, respectively. The differences between the average C_T_ of the LOD and NTC were >4 cycles in all tested probes, which indicates nontemplate-specific products of <6.25%.

### Association analysis

Association of the NGS58879 SNP marker genotype with the bulls’ breeding values for fertility was analyzed by the PLINK linear/logistic regression option (Purcell *et al.* 2007). The population included 900 sires, including the 10 sires tested by the daughter design. A regression analysis of 326 sires’ breeding values for fertility on the overall CNV or specific variant copy number was performed using the “fit Y by X” option of the JMPIN 5.0.1a statistical software (SAS Institute Inc). In addition, multiple regression was performed for the sires’ breeding values for fertility with NGS58879 and V_1_ as independent variables.

## Results

### Concordance and effects associated with NGS-58779

Of the 704 SNPs in the QTL CI on BTA7 that was tested for concordance, only a single intergenic SNP, NGS-58779 positioned at 4922643 bp (Btau 4.0 genome assembly), showed apparent full concordance; all five patriarchs that were heterozygous for the QTL were heterozygous for the marker, and the remaining five sires, homozygous for the QTL, were homozygous for the marker. The “A” allele of the NGS-58779 was associated with the positive haplotype of the fertility QTL identified in the daughter design analysis in four of the five heterozygous sires, and corresponded to the effect associated with the marker in the general sire population. Thus concordance was obtained in 9 out of 10 families.

The NGS-58779 marker showed a significant association to breeding values of sires for fertility in a sample of 900 sires (*P* ≤ 1.5*10^−6^). The frequency of the “A” allele, associated with increased fertility, was 31.5%. The observed allele substitution effect was 0.57 trait units.

### Haplotype phasing analysis

The neighboring genomic region of the concordant SNP was analyzed in order to explore the linkage disequilibrium (LD) boundaries of the QTL region. Although the Plink haplotype phase analysis was performed without including relationships among bulls, no conflicts were found between the Plink and LSPH results. All chromosomes carrying the “A” allele of NGS-58779 shared a common haplotype extending from 4.65 to 4.92 Mbp (Table 3).

**Table 3:** Phased haplotypes of the 10 analyzed sires

Sire	QTL*a*	Haplotypes (SNPs Position on BTA7 in Mbp)																			
		4.43	4.50	4.52	4.59	4.63	4.65	4.67	4.72	4.76	4.82	**4.92**	4.96	5.01	5.03	5.06	5.09	5.11	5.14	5.16	5.20
2278	+	G	A	G	G	A	**G**	**A**	**A**	**G**	**G**	**A**	G	G	A	G	G	G	A	A	G
		G	G	C	A	A	G	A	A	G	G	G	G	G	A	G	G	G	A	A	G
3070	+	G	G	G	G	G	**G**	**A**	**A**	**G**	**G**	**A**	A	G	A	A	G	G	A	G	G
		G	G	G	G	G	G	A	A	G	G	G	A	G	A	G	G	G	G	G	G
3089	+	G	G	G	G	G	**G**	**A**	**A**	**G**	**G**	**A**	A	G	G	G	G	C	G	A	G
		G	G	G	G	G	G	A	A	G	A	G	G	G	A	G	G	G	A	A	G
3208	+	G	G	G	G	G	**G**	**A**	**A**	**G**	**G**	**A**	A	G	G	G	G	C	G	A	G
		G	G	G	G	G	G	A	A	G	G	G	G	A	G	A	G	G	A	G	A
3258	+	G	A	G	G	G	**G**	**A**	**A**	**G**	**G**	**A**	G	G	A	G	G	G	G	G	G
		G	G	G	G	G	G	A	A	G	G	G	G	A	G	G	G	G	A	G	G
3099	−	G	G	G	G	G	**G**	**A**	**A**	**G**	**G**	**A**	A	G	G	G	G	C	G	A	G
		G	A	G	G	G	**G**	**A**	**A**	**G**	**G**	**A**	G	G	A	G	G	G	G	G	G
2357	−	G	G	G	G	G	**G**	**A**	**A**	**G**	**G**	**A**	A	G	G	G	G	C	G	A	G
		G	G	G	G	G	**G**	**A**	**A**	**G**	**G**	**A**	G	G	A	G	G	G	G	G	G
2283	−	G	G	G	G	G	G	A	A	G	A	G	G	G	A	G	G	G	A	G	G
		A	G	G	G	G	G	A	A	G	G	G	G	A	G	G	G	C	G	A	G
3241	−	G	G	G	G	G	G	A	A	G	G	G	G	A	G	A	G	G	A	G	G
		A	G	G	G	G	G	A	A	G	G	G	G	A	G	G	G	C	G	G	A
3274	−	G	G	G	G	G	G	A	A	G	G	G	G	A	G	A	G	G	A	G	A
		G	A	G	G	A	G	G	G	G	G	G	G	G	A	G	A	G	A	G	G

The NGS-58779 SNP in bold is located at 4.92 Mbp. The common sub-haplotype associated with the NGS-58779 “A” allele is in bold.

^a^ QTL segregation status of sires (+ for heterozygous and − for homozygous).

### Comparative mapping and positional cloning

**Figure 1:**
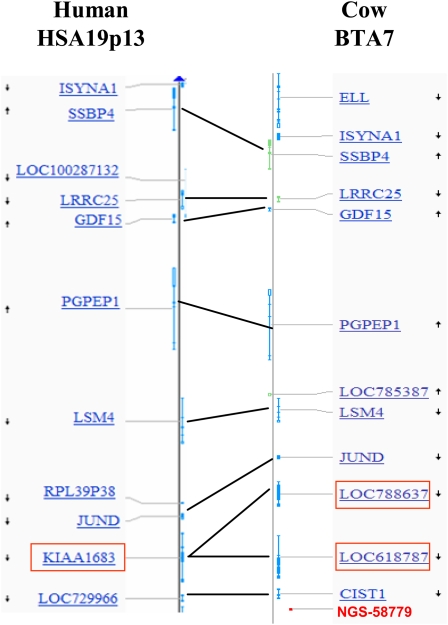
Bovine/human comparative mapping of the QTL region: lines connect the orthologous genes. The human *KIAA1683* gene and the bovine orthologous duplication (*LOC788637* and *LOC618787*) are marked in red. The SNP marker NGS-58879 is in the bottom of the BTA7 map and appears in red.

A comparative map of the bovine common haplotype region and its human orthologous region (HSA19p13) is shown in Figure 1. The region contains 11 genes (*CIST1*, *JUND*, *LSM4*, *GDF15*, *KIAA1683*, *LOC785387*, *PGPEP1*, *LRRC25*, *SSBP4*, *ISYNA1*, and *ELL*) in the bovine gene map. Partial sequencing of the exons of four genes (*CIST1*, *JUND*, *LSM4*, and *GDF15*) did not identify a complete concordance between any of the polymorphisms and the QTL status of the patriarchs (Table 4).

**Table 4:** Partial sequencing of positional genes

Gene	Number of Exons		Sequencing Size (bp)	Polymorphism Type*a*	Location in Gene	Genomic Location*b*	Concordance*c*
	Total	Sequenced					
*GDF15*	2	2	1067	A to T non-syn T/S	Exon 2	4,704,137	3/4
*LSM4*	5	2	486	—			0
*JUND*	1	1	302	—			0
*KIAA1683*	6	6	3586	37 SNPs			9/10
*CIST1*	4	4	1623	T to C non-syn V/A	Exon 1	4,911,890	4/5
				C to T non-syn T/M	Exon 2	4,914,545	9/10
				C to A non-syn L/M	Exon 2	4,914,833	8/10
				A to G 3′ UTR	Exon 4	4,916,672	0/3
				T to G	Intron 12	4,920,670	3/5

^a^ Non-syn stands for nonsynonymous mutation that is capable of encoding amino acid substitution. UTR stands for untranslated region.

^b^ Genomic locations of the identified SNP on BTA7 in bp (Btau4.0).

^c^ Concordance of polymorphism with the segregation status of sires for the QTL.

**Figure 2:**
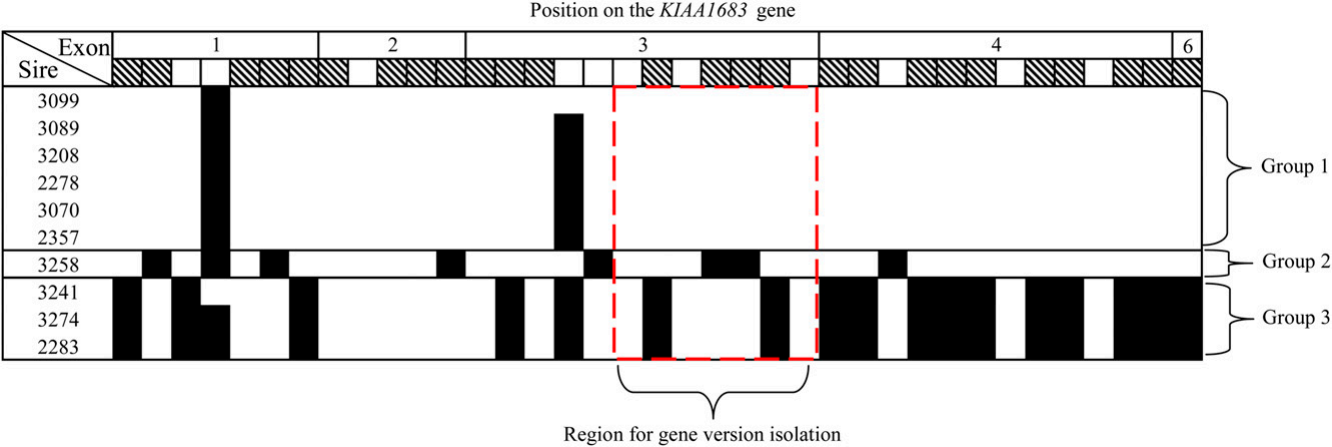
Representation of the polymorphic sites found among the 10 sires along exons 1 to 6 of the *KIAA1683* genes: Striped sites indicate the location of differences between the two gene duplicates in the reference genome. Sites in which only a single nucleotide was found for the individual sires are marked in black. The patterns of the 10 sires are clustered into three major groups: 1) “heterozygous” in >95% of the sites (3099, 2357, 3089, 3208, 3070, and 2278); 2) “homozygous” in 8 sites (3258) and 3) “homozygous” in the same 16 sites (2283, 3241, and 3274). The red dashed line indicates the section of exon 3 which was chosen as a target for the gene variants isolation. This region distinguishes between the three cluster groups with at least two unique homozygous sites.

The comparative map showed duplication in the bovine *KIAA1683* orthologous genes (*LOC788637* and *LOC6181787*). Alignment analysis demonstrated very high similarity in the sequence of both genes (∼95% identity). Their intron 1 contains a duplication of a section of the exon 1 sequence. *LOC788637* has three copies of the exon 1 sequence, while *LOC6181787* has only two. Two repetitive regions are present in intron 4 of both gene duplicates. Sequencing analysis of the exons regions revealed 37 sites of polymorphism between the gene variants, 26 of which were predicted to be due to the differences between the two paralogues in the reference genome (Btau 4.0). Figure 2 summarizes the polymorphism patterns among the 10 sires for exons 1 to 6 (the full genotyping report is summarized in supporting information, Table S1). These patterns may be clustered into three major groups: 1) “heterozygous” in >95% of the sites (sires 3099, 2357, 3089, 3208, 3070, and 2278); 2) “homozygous” in 8 sites (sire 3258) and 3) “homozygous” in the same 16 sites (sires 2283, 3241, and 3274).

### Isolation of *KIAA1683* variants

The sequencing analysis revealed 11 novel polymorphism sites in the *KIAA1683* gene that were not found in the two reported gene variants. This indicated that there are additional variants of the gene. In order to isolate theses putative variants we focused on exon 3, which showed seven polymorphic sites, including four informative sites that differentiate the three patterns found in the sample of sires (Figure 2).

**Figure 3:**
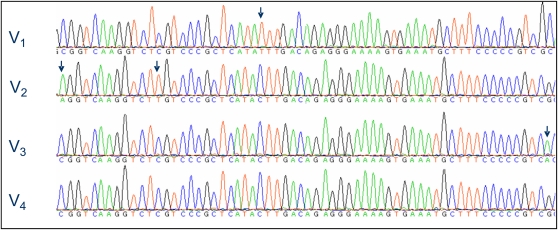
Sequencing chromatograms of four different *KIAA1683* variants. A 60-bp sequence within the *KIAA1683* third exon is displayed. Arrows mark specific nucleotide changes that differentiate the gene variants.

We used two allele specific primers that selectively amplified the A/G alleles of the 4856941/4896528 polymorphism site (SP_1_ and SP_2_, Table 1). All sires amplified both alleles, and the resulting upstream sequence polymorphism is presented in Table 5. The polymorphism of cluster group 3 (sires 2283, 3241, and 3274) was different from that of the other sires, showing only two distinct variants of the *KIAA1683* exon 3 region (V_2_ by SP_2_ and V_3_ by SP_1_). The polymorphism of the other sires upstream to the A/G site was complex, indicating the existence of additional gene variants. The SP_2_ primer enabled us to identify two distinct variants of the gene: V_2_ (isolated in sires 2278, 3070, 3089, 3208, 2357, 3241, 3274, and 2283), V_4_ (isolated in 3258) and both variants in 3099. The SP_1_ primer enabled us to isolate V_3_ in sires 2283, 3241, and 3274. The SP_1_ sequence of the other sires was identical, indicating a common gene version in addition to V_3_. This expected gene version (V_1_) is identical to the *LOC788637* reference genome sequence. Thus, four different variants of the bovine *KIAA1683* exon 3 were identified (Table 6, Figure 3). BLAST analysis of the variants identified perfect matches for the V_1_ and V_2_ variants in the bovine reference genome. V_3_ was found by BLAST analysis against the bovine HTGS sequence database, while V_4_ is novel (Table 6, Figure 3).

**Table 5:** Allele-specific amplification and polymorphism of the 10 sires

Primer	Sire	Cluster Group*b*	Polymorphism on BTA7 (in bp)*a*							Gene Variant
			**4856941** **4896528**	4857096 4896683	4857126 4896713	4857153 4896740	4857165 4896752	4857178 4896765	4857214 4896801	
SP_1_	2278		A	C/T	C/T	C	C	T/C	G/A	V_3_, V_1_
	3070		A	C/T	C/T	C	C	T/C	G/A	V_3_, V_1_
	3089	1	A	C/T	C/T	C	C	T/C	G/A	V_3_, V_1_
	3208		A	C/T	C/T	C	C	T/C	G/A	V_3_, V_1_
	2357		A	C/T	C/T	C	C	T/C	G/A	V_3_, V_1_
	3099		A	C/T	C/T	C	C	T/C	G/A	V_3_, V_1_
	3258	2	A	C/T	C/T	C	C	T/C	G/A	V_3_, V_1_
	3241		A	C	T	C	C	C	A	V_3_
	3274	3	A	C	T	C	C	C	A	V_3_
	2283		A	C	T	C	C	C	A	V_3_
SP_2_	2278		G	C	C	A	T	C	G	V_2_
	3070		G	C	C	A	T	C	G	V_2_
	3089	1	G	C	C	A	T	C	G	V_2_
	3208		G	C	C	A	T	C	G	V_2_
	2357		G	C	C	A	T	C	G	V_2_
	3099		G	C	C	C/A	T/C	C	G	V_2_, V_4_
	3258	2	G	C	C	C	C	C	G	V_4_
	3241		G	C	C	A	T	C	G	V_2_
	3274	3	G	C	C	A	T	C	G	V_2_
	2283		G	C	C	A	T	C	G	V_2_

^a^ Location of the polymorphic sites on the *KIAA1683* exon 3 in BTA7 on the Btau4.0 genome assembly; positions for each polymorphism are shown for *LOC788637* and *LOC618787* loci, on the upper and lower lines, respectively. The SNP location targeted by the allele-specific primers is in bold.

^b^ The polymorphism-pattern cluster of the sires according to their polymorphic pattern (Figure 2).

**Table 6:** Isolated *KIAA1683* exon 3 variants

Gene Variant	Reference ID	SNP Location on BTA7 (in bp)*a*						
		4856941 4896528	4857096 4896683	4857126 4896713	4857153 4896740	4857165 4896752	4857178 4896765	4857214 4896801
V_1_	*LOC788637*	A	T*b*	C	C	C	T	G
V_2_	*LOC618787*	G	C	C	A*c*	T	C	G
V_3_	ti109805135	A	C	T*d*	C	C	C	A
V_4_	—	G	C	C	C	C	C	G

^a^ Location of polymorphic sites on the Btau4.0 genome assembly; positions are shown for the *LOC788637* and the *LOC618787* loci, on the upper and lower lines, respectively.

^b^ Polymorphism site used by qPCR to selectively amplify V_1_.

^c^ Polymorphism site used by qPCR to selectively amplify V_2_.

^d^ Polymorphism site used by qPCR to selectively amplify V_3_.

### Copy number analysis and association with breeding values for fertility

**Figure 4:**
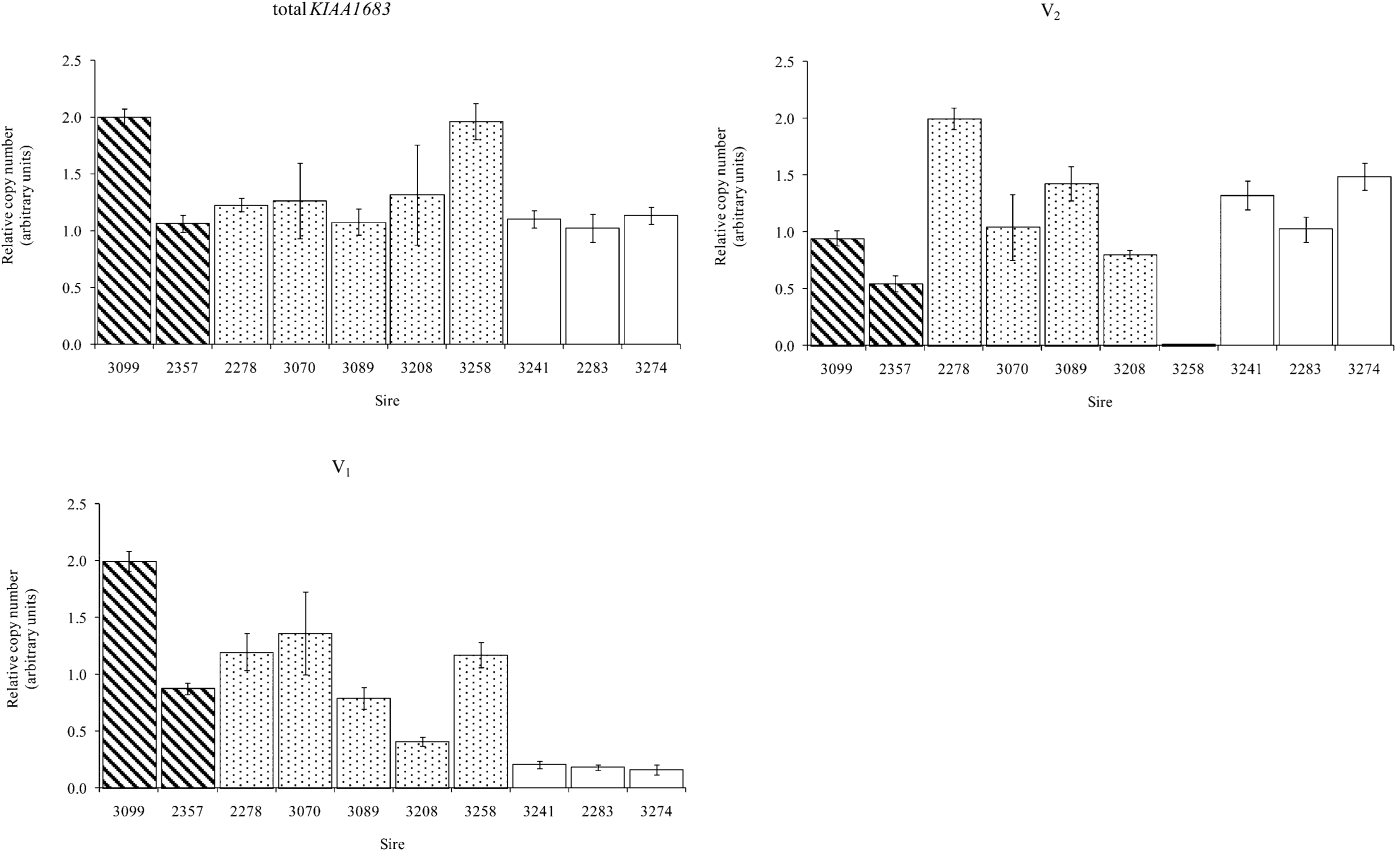
Normalized relative copy number (arbitrary units) of total *KIAA1683*, V_1_ and V_2_ of the 10 sires with known QTL genotypes: QTL genotypes were determined by the daughter design; dotted bars denote the heterozygous sires. Genotypes of the NGS-58779 SNP “AA”, “AG” and “GG” are indicated by striped, dotted, and blank bars, respectively. CNV was analyzed by qPCR and quantities were normalized to *RPP30*, an autosomal non-CNV reference gene. Values were normalized to the highest value of sire which was assigned the value of 2 (arbitrary units). Error bars indicates the standard error between the qPCR replicates.

**Figure 5:**
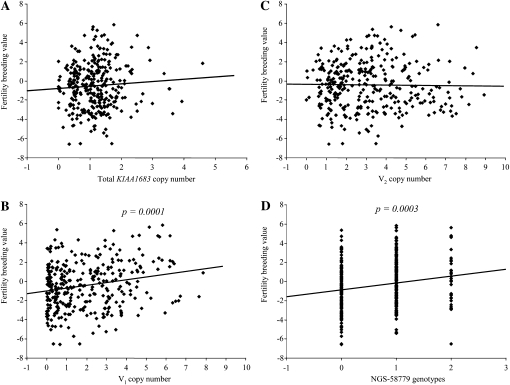
CNV association test: Linear regressions of sires’ breeding values for fertility on copy number of total *KIAA1683* (A), V_1_ (B),V_2_ (C), and genotypes of NGS-58779 (D). NGS-58779 genotypes were scored as 0, 1, and 2 for homozygotes (G), heterozygotes (AG), and homozygotes (A), respectively. Probabilities are given for the significant regressions.

The relative copy number of the 10 sires for *KIAA1683*, and its V_1_ and V_2_ variants were estimated using qPCR (Figure 4). The results confirm the genotyping results using the variant specific analysis. Sire 3258 which lacks V_2_, and sires 2283, 3241, and 3274 which lack V_1_ showed only residual copy number reflecting the absence of these gene variants. The regressions of breeding values of 326 sires for fertility on their copy number for total *KIAA1683* and V_2_ were not significant (Figure 5, A and C). On the other hand, a highly significant regression was found for V_1_ (*P* < 0.0001; R^2^ = 0.047, Figure 5B) which is similar to the association of the NGS-58779 SNP marker using the same population structure (*P* < 0.0003; R^2^ = 0.037, Figure 5D). When both NGS-58779 and V_1_ copy number were included in the model, both factors were significantly associated with fertility (*P* < 0.01 and *P* < 0.03, respectively), increasing the R^2^ to 0.069.

## Discussion

### Concordance testing

In cattle, numerous genome scans were conducted during the past 15 years to identify QTL. These scans, based either on daughter of granddaughter designs (Weller *et al.* 1990), led to the identification of many chromosomal regions with effects on nearly all of the economically important traits (http://www.genome.iastate.edu/cgi-bin/QTLdb/BT/index). However, the CI for QTL location is generally >20 cM, thus containing hundreds of genes. Increasing marker density beyond one marker per 10 cM has very limited effect on the length of the CI of the QTL (Ron and Weller 2007). The identification of a putative QTN requires full concordance with the QTL status of individuals. Such information can only be detected by pedigree analysis, such as daughter or granddaughter designs. The probability of obtaining full concordance by chance depends on the number of individuals with known QTL status analyzed, the number of segregating individuals (heterozygous for the QTL), and the number of markers tested (Ron and Weller 2007).

The 27 cM 95% CI interval of the QTL for female fertility in the centromeric region of BTA7 (Weller *et al.* 2008) contains 704 SNPs from the Illumina BovineSNP50 BeadChip. Of these, only a single intergenic SNP, NGS-58779, showed nearly complete concordance (9 out of 10 families). The probability to obtain such concordance by chance is 0.000152 (see *Materials and Methods*). By accounting for multiple testing of 704 SNPs only 0.1 SNP is expected to achieve this concordance level by chance. Furthermore, a highly significant effect on female fertility was associated with this marker in the sample of 900 sires genotyped (*P* < 1.5*10^−6^).

### Common haplotype identification

Haplotype phase analysis for SNP markers within the flanking 0.5 Mbp upstream and downstream to NGS-58779 interval showed that all chromosomes carrying the “A” allele of NGS-58779 shared a common haplotype extending from 4.65 to 4.92 Mbp (Table 3). However, because the frequencies of the SNPs alleles included in this haplotype were >0.8, this haplotype was also found in some of the chromosomes carrying the “G” allele of NGS-58779. On the other hand, no common haplotype associated with the “A” allele was discernable on the other side of NGS-58779. We hypothesize that this common haplotype region contains the causative mutation for this QTL. Cohen-Zinder *et al.* (2005) identified a common haplotypes among the two segregating sires for the QTL for milk protein on BTA6. The QTN was eventually identified in *ABCG2*, one of the five genes in this haplotype region.

### Polymorphism in the *KIAA1683* gene

Comparative mapping of the common haplotype region to the human orthologous region on HAS19 revealed duplication in the bovine *KIAA1683* gene (*LOC788637* and *LOC6181787*). The two gene duplicates shared high sequence similarity (∼95%). Among the genes in the region that were sequenced, only *KIAA1683* revealed a high number of polymorphic sites that may indicate copy number variation. Twenty six sites were attributed to the differences between the two reference genome gene duplicates. The additional 11 sites indicated the possibility for more unidentified gene variants. The polymorphic patterns identified among the 10 sires, which clustered into three major groups, suggest that there are differences in the gene variants held by the different sires. The gene variants isolation analysis confirmed our assumption. A summary of the gene variants harbored by the 10 sires is presented in Table 7. The three sires homozygous for the NGS-58779 “G” allele—2283, 3241, and 3274—share variants V_2_ and V_3_. The two sires homozygous for the NGS-58779 “A” allele—3099 and 3257—also have variant V_1_. Sire 3099 also has variant V_4_. All four sires that are heterozygous for both the fertility QTL and the NGS-58779 marker—2278, 3070, 3089 and 3208—have variants V_1_, V_2_, and V_3_. Sire 3258, which is heterozygous for the QTL, but shows opposite allelic association between the NGS-58779 and the QTL alleles, has a unique pattern of variants: V_1_, V_3_, and V_4_.

**Table 7:** Summary of the *KIAA1683* gene variants harbored by the 10 sires

Sire	Gene Variant				NGS-58779*a*	QTL*b*
	V_1_	V_2_	V_3_	V_4_		
3099	+	+	+	+	A/A	−
2357	+	+	+	−	A/A	−
3089	+	+	+	−	A/G	+
3208	+	+	+	−	A/G	+
2278	+	+	+	−	A/G	+
3070	+	+	+	−	A/G	+
3258	+	−	+	+	A/G	+
3241	−	+	+	−	G/G	−
3274	−	+	+	−	G/G	−
2283	−	+	+	−	G/G	−

^a^ Genotypes of the NGS-58779 SNP.

^b^ QTL segregation status of the sires (+ for heterozygous and − for homozygous).

The gene variants’ patterns of the 10 sires may indicate the presence of a CNV, as the sires varied not only in the type of gene variants, but also in their number (from two to four). The qPCR analysis revealed a CNV not only in the total *KIAA1683* but also in V_1_ and V_2_ specific gene variants.

Previous work identified segmental duplication and CNV in this genomic region. Bae *et al.* (2010) used the BovineSNP50 BeadChip signal intensity to identify CNV region in Korean cattle. They identified a CNV region that spans BTA7: 4.65–5.03 Mbp, which overlaps with our common haplotype region (BTA7: 4.65-4.92 Mbp). Liu *et al.* (2009) performed a systematic computational genome-wide analysis of segmental duplications in cattle based on identifying paralogous sequences ≥1 kb in length with ≥90% sequence identity, and genomic regions that exhibit significant increase in depth of coverage in the whole genome shotgun sequences. The analysis identified significant peaks in both *KIAA1683* genes indicating segmental duplication. In contrast to Bae *et al.* (2010), this analysis indicated that the segmental duplication in this region is limited only to the *KIAA1683* genes.

### *KIAA1683* Copy number analysis

Of the four identified *KIAA1683* gene variants identified by the allele specific PCR and sequencing, only V_1_ indicated an association with the NGS-58779 marker in the 10 sires analyzed. The sires that were homozygous for the NGS-58779 “G” allele (2283, 3241, and 3274) lack V_1_, while the sires homozygous for the “A” allele (3099 and 3257) and the heterozygous sires (2278, 3070, 3089, 3208, and 3258) all have V_1_ (Table 7). In recent years, CNV has been increasingly recognized as a major source of heritable variation that impacts complex traits (Redon *et al.* 2006). Thus, we quantified the copy number of total *KIAA1683* and its variants, V_1_ and V_2_. Only V_1_ copy number showed a significant association with fertility in the general population (*P* < 0.0001). It appears that copy number variation of different gene variants might be considered as independent genetic markers for association analyses. A similar phenomenon was reported for the human *CYP2D* locus located on chromosome 22 (HSA22q12). *CYP2D* is an enzyme expressed in the human liver. The *CYP2D* locus consists of three tandem repeats of homologous sequences of which *CYP2D6* is the functional gene, while *CYP2D7* and *CYP2D8* are pseudogenes. Apparently only CNV of *CYP2D6* was associated with drug metabolism phenotype (Abraham *et al.* 2010; Ingelman-Sundberg 2004). Analogously, it is possible that V_2_ to V_4_ variants of *KIAA1683* are pseudogenes, while V_1_ variant is the only active gene. This hypothesis may be tested by gene expression analysis which will identify the gene variants that are actually being transcribed to mRNA in different bovine tissues. Expression data for *LOC788637* and *LOC6181787* genes were searched in the bovine gene atlas but no informative results were found (Harhay *et al.* 2010, http://bovineatlas.msstate.edu/).

Inclusion of both NGS-58779 and V_1_ copy number in the analysis model yielded statistical significance for both factors and increased the R^2^. It is expected that when a causal mutation is included in the analysis model with additional linked markers, only the causal mutation will be significant (Ron and Weller 2007). Thus both factors contribute to the explained variation, and apparently neither of them is the causal mutation. It should be noted that *CIST1* also showed high concordance between the polymorphism and the segregation status of sires for the QTL.

## Conclusions

We demonstrate a new strategy for fine mapping QTL which combines GWAS data together with daughter design results and concordance testing. We identified a single intergenic SNP, NGS-58779, which showed concordance for 9 out of 10 sires, and a highly significant association with fertility in the population. We further identified a common haplotype associated with the rare favorable allele of the marker, from 4.65 to 4.92 Mbp on BTA7. Thus, the 27 cM CI of the fertility QTL was reduced to a 270 kbp region which contains only 11 genes that should be considered as positional candidates for the identification of the causative quantitative trait nucleotide.

An analysis of the CNV in the *KIAA1683* gene showed that only the number of copies of a specific gene variant (V_1_) was significantly associated with breeding values of sires for fertility. Thus quantifying the copy number of different gene variants is of paramount importance for association analyses.

## Supplementary Material

Supporting Information

## References

[bib1] Abraham J. Maranian M. Driver K. Platte R. Kalmyrzaev B., 2010 *Cyp2d6* gene variants: Association with breast cancer specific survival in a cohort of breast cancer patients from the united kingdom treated with adjuvant tamoxifen. Breast Cancer Res. 12: R64 20731819 10.1186/bcr2629 PMC2949659

[bib2] Bae J. S. Cheong H. S. Kim L. H. NamGung S. Park T. J., 2010 Identification of copy number variations and common deletion polymorphisms in cattle. BMC Genomics 11: 232 20377913 10.1186/1471-2164-11-232 PMC2859865

[bib3] Baruch E. Weller J. I. Cohen-Zinder M. Ron M. Seroussi E., 2006 Efficient inference of haplotypes from genotypes on a large animal pedigree. Genetics 172: 1757–1765 16361242 10.1534/genetics.105.047134 PMC1456282

[bib4] Cohen-Zinder M. Seroussi E. Larkin D. M. Loor J. J. Wind A. E.-d., 2005 Identification of a missense mutation in the bovine *ABCG2* gene with a major effect on the QTL on chromosome 6 affecting milk yield and composition in Holstein cattle. Genome Res. 15: 936–944 15998908 10.1101/gr.3806705 PMC1172037

[bib5] D'Haene B. Vandesompele J. Hellemans J., 2010 Accurate and objective copy number profiling using real-time quantitative PCR. Methods 50: 262–270 20060046 10.1016/j.ymeth.2009.12.007

[bib6] Fadista J. Thomsen B. Holm L.-E. Bendixen C., 2010 Copy number variation in the bovine genome. BMC Genomics 11: 284 20459598 10.1186/1471-2164-11-284 PMC2902221

[bib7] Gonzalez-Recio O. Alenda R. Chang Y. M. Weigel K. A. Gianola D., 2006 Selection for female fertility using censored fertility traits and investigation of the relationship with milk production. J. Dairy Sci. 89: 4438–4444 17033033 10.3168/jds.S0022-0302(06)72492-4

[bib8] Harhay G. Smith T. Alexander L. Haudenschild C. Keele J., 2010 An atlas of bovine gene expression reveals novel distinctive tissue characteristics and evidence for improving genome annotation. Genome Biol. 11: R102 20961407 10.1186/gb-2010-11-10-r102 PMC3218658

[bib9] Ingelman-Sundberg M., 2004 Genetic polymorphisms of Cytochrome P450 2d6 (*CYP2d6*): Clinical consequences, evolutionary aspects and functional diversity. Pharmacogenomics J. 5: 6–13 15492763 10.1038/sj.tpj.6500285

[bib10] Kuhn M. T. Hutchison J. L. Wiggans G. R., 2006 Characterization of Holstein heifer fertility in the united states. J. Dairy Sci. 89: 4907–4920 17106123 10.3168/jds.S0022-0302(06)72541-3

[bib11] Liu G. Ventura M. Cellamare A. Chen L. Cheng Z., 2009 Analysis of recent segmental duplications in the bovine genome. BMC Genomics 10: 571 19951423 10.1186/1471-2164-10-571 PMC2796684

[bib12] Liu G. E. Hou Y. Zhu B. Cardone M. F. Jiang L., 2010 Analysis of copy number variations among diverse cattle breeds. Genome Res. 20: 693–703 20212021 10.1101/gr.105403.110 PMC2860171

[bib13] Ma R. Z. Beever J. E. Da Y. Green C. A. Russ I., 1996 A male linkage map of the cattle (*bos taurus*) genome. J. Hered. 87: 261–271 8776874 10.1093/oxfordjournals.jhered.a022999

[bib14] Purcell S. Neale B. Todd-Brown K. Thomas L. Ferreira M. A., 2007 Plink: A tool set for whole-genome association and population-based linkage analyses. Am. J. Hum. Genet. 81: 559–575 17701901 10.1086/519795 PMC1950838

[bib15] Redon R. Ishikawa S. Fitch K. R. Feuk L. Perry G. H., 2006 Global variation in copy number in the human genome. Nature 444: 444–454 17122850 10.1038/nature05329 PMC2669898

[bib16] Ron M. Weller J., 2007 From QTL to QTN identification in livestock - winning by points rather than knock-out: A review. Anim. Genet. 38: 429–439 17697134 10.1111/j.1365-2052.2007.01640.x

[bib17] Seroussi E. Glick G. Shirak A. Yakobson E. Weller J., 2010 Analysis of copy loss and gain variations in Holstein cattle autosomes using beadchip SNPs. BMC Genomics 11: 673 21114805 10.1186/1471-2164-11-673 PMC3091787

[bib18] Staden R. Beal K. F. Bonfield J. K., 1999 The Staden package, 1998. Methods Mol. Biol. 132: 115–130 10547834 10.1385/1-59259-192-2:115

[bib19] Veerkamp R. F. Koenen E. P. C. De Jong G., 2001 Genetic correlations among body condition score, yield, and fertility in first-parity cows estimated by random regression models. J. Dairy Sci. 84: 2327–2335 11699466 10.3168/jds.S0022-0302(01)74681-4

[bib20] Wang J. Ramakrishnan R. Tang Z. Fan W. Kluge A., 2010 Quantifying *EGFr* alterations in the lung cancer genome with nanofluidic digital PCR arrays. Clin. Chem. 56: 623–632 20207772 10.1373/clinchem.2009.134973

[bib21] Weller J. I. Ezra E., 1997 Genetic analysis of somatic cell score and female fertility of Israeli Holsteins with an individual animal model. J. Dairy Sci. 80: 586–593 9098810 10.3168/jds.S0022-0302(97)75974-5

[bib22] Weller J. I. Golik M. Reikhav S. Domochovsky R. Seroussi E., 2008 Detection and analysis of quantitative trait loci affecting production and secondary traits on chromosome 7 in Israeli Holsteins. J. Dairy Sci. 91: 802–813 18218768 10.3168/jds.2007-0367

[bib23] Weller J. I. Kashi Y. Soller M., 1990 Power of daughter and granddaughter designs for determining linkage between marker loci and quantitative trait loci in dairy cattle. J. Dairy Sci. 73: 2525–2537 2258496 10.3168/jds.S0022-0302(90)78938-2

[bib24] Weller J. I. Glick G. Ezra E. Zeron Y. Seroussi E., 2010 Paternity validation and estimation of genotyping error rate for the bovinesnp50 beadchip. Anim. Genet. 41: 551–553 20331599 10.1111/j.1365-2052.2010.02035.x

