# Supplementary material for: Fine Mapping of a QTL for Fertility on BTA7 and Its Association With a CNV in the Israeli Holsteins
Source: G3 (Bethesda). 2011 Jun 1;1(1):65–74. doi: 10.1534/g3.111.000299 (PMC3276122; doi:10.1534/g3.111.000299)
Supplement: Supporting Information [file supp_1_1_65__index.html]

Supporting Information 

# Fine Mapping of a QTL for Fertility on BTA7 and Its Association With a CNV in the Israeli Holsteins

## Supporting Information for Glick *et al.*, 2011

**Files in this Data Supplement:**

- Table S1 - KIAA1683  full  polymorphism  report  (Microsoft Excel, .xls, 32 KB)
